# Adaptive Tip Selection for DAG-Shard-Based Federated Learning with High Concurrency and Fairness

**DOI:** 10.3390/s25010019

**Published:** 2024-12-24

**Authors:** Ruiqi Xiao, Yun Cao, Bin Xia

**Affiliations:** School of Computer Science, Nanjing University of Posts and Telecommunications, Nanjing 210023, China; b22131007@njupt.edu.cn (R.X.); 1223045605@njupt.edu.cn (Y.C.)

**Keywords:** directed acyclic graph, blockchain, federated learning, high concurrency, fair incentive

## Abstract

To cope with the challenges posed by high-concurrency training tasks involving large models and big data, Directed Acyclic Graph (DAG) and shard were proposed as alternatives to blockchain-based federated learning, aiming to enhance training concurrency. However, there is insufficient research on the specific consensus designs and the effects of varying shard sizes on federated learning. In this paper, we combine DAG and shard by designing three tip selection consensus algorithms and propose an adaptive algorithm to improve training performance. Additionally, we achieve concurrent control over the scale of the directed acyclic graph’s structure through shard and algorithm adjustments. Finally, we validate the fairness of our model with an incentive mechanism and its robustness under different real-world conditions and demonstrate DAG-Shard-based Federated Learning (DSFL)’s advantages in high concurrency and fairness while adjusting the DAG size through concurrency control. In concurrency, DSFL improves accuracy by 8.19–12.21% and F1 score by 7.27–11.73% compared to DAG-FL. Compared to Blockchain-FL, DSFL shows an accuracy gain of 7.82–11.86% and an F1 score improvement of 8.89–13.27%. Additionally, DSFL outperforms DAG-FL and Chains-FL on both balanced and imbalanced datasets.

## 1. Introduction

As we progress into the era of big data and Web 3.0, the volume of data is increasing dramatically, making centralized AI training potentially unsuitable [1]. Distributed learning is proposed to address issues related to large data volumes and high data transmission costs. To address the security concerns regarding data and training and the lack of data volume, Federated Learning (FL) has been proposed. However, the traditional server and device architecture used in FL also faces challenges, such as single points of failure and a lack of client trust in data privacy protection. Following the introduction of the General Data Protection Regulation (GDPR), growing concern exists regarding data privacy during training. In this context, blockchain technology, characterized by transparency, immutability, and high scalability, can serve as an architecture for FL. Leveraging the traceability features of blockchain better identifies the participation of malicious client nodes, thereby enhancing the overall stability of the training process.

Each block in the blockchain is connected to the previous block through a hash function, forming a chain structure. It is tough to tamper with the contents of a blockchain, which requires more than half of the nodes, so blockchains have good structural properties. Although blockchain is considered a promising solution that can transform FL training in a decentralized manner and improve security during training [2], Blockchain-FL still has many shortcomings, such as concurrency, anti-interference, and incentive mechanisms.

### 1.1. High Concurrency

The most prominent disadvantage of Blockchain-FL is its efficiency. While single-chain hashed blockchains are stable and resistant to double-spending problems, scalability presents a significant challenge. The Bitcoin block size is currently limited to 1 MB, with a block being mined approximately every 10 min. Additionally, the time efficiency of consensus mechanisms, such as Proof of Work (PoW) and Proof of Stake (PoS), used in blockchain packaging is low. The effectiveness of peer-to-peer (P2P) systems may not adequately respond to large-scale node interactions quickly, leading to lower efficiency in consensus promotion. This limitation hinders the ability to meet the demands for timely and effective training results from a large volume of federated data samples during short training intervals.

Although cross-chain interactions in blockchain can assist in global model aggregation to some extent, frequent cross-chain activities introduce complexities in permission authentication, consensus design, and high communication costs. Limited resources, high network throughput, latency, and the time costs associated with blockchain consensus hinder the potential for single-chain structures to achieve effective results quickly.

Directed Acyclic Graph (DAG)-based blockchain is more suitable for implementing large-scale FL in mobile wireless networks. DAG-based blockchain networks can save significant resources since they do not require complex PoW execution. However, the efficiency of clients directly interacting with the DAG blockchain for FL is relatively low in large-scale DAG networks. Therefore, traditional DAG-FL should still be improved in terms of concurrency. Current FL updates propagate across the nodes for aggregation, which could lead to notable delays and inefficiencies. Consequently, a bottleneck limits the scalability of the system [3].

### 1.2. Anti-Interference

In recent years, data privacy protection has become a widely discussed issue. Ensuring the security and privacy of vast amounts of non-independent, non-orthogonal, and imbalanced client data has become an increasingly important issue. Unauthorized data distribution and storage are particularly concerning [4]. In many fields, such as healthcare and finance, customers are reluctant to share their data due to the sensitivity of the information [5]. Traditional centralized learning is becoming less suitable, yet collecting data to address problems remains necessary. Therefore, due to the decentralized architecture of FL, it has gained widespread adoption. Edge devices can train and upload weights locally to ensure the reliability and security of data.

However, the risk of privacy breaches from network attacks persists during the weight upload. Existing research indicates that client data can be recovered by stealing weights. Consequently, integrating blockchain technology with its immutability, traceability, and transparency can effectively mitigate this issue [6]. By incorporating technologies such as Wasserstein Generative Adversarial Networks (WGANs), Differential Privacy (DP), and Homomorphic Encryption (HE) [7] into Blockchain-FL, its resistance to interference can be significantly enhanced. However, the existing methods still have limitations, such as the high complexity of HE and the potential impact of DP noise on model performance. Lu et al. proposed a class-imbalanced privacy-preserving federated learning framework for the fault diagnosis of a decentralized wind turbine [8]. They employed a biometric authentication technique to ensure that only legitimate entities can access private data and defend against malicious attacks. However, validating malicious entities may unintentionally target clients with low model accuracy or limited computational power, who are clearly not acting maliciously. To encourage more clients to join rather than deter them, our DAG-Shard-based Federated Learning (DSFL) uses FL technology to protect privacy and implements a three-layer defense mechanism to guard against malicious nodes. The first line of defense is an initial review by each shard’s internal auditing committee. We propose separate consensus mechanisms for large and small shards to ensure fair shard leader elections. Shard leaders can detect useless models and malicious nodes, preventing them from polluting the DAG blockchain and wasting resources. The second line of defense is the DAG committee, formed by shard leaders, which balances global model development and blocks compromised shard models from being submitted on-chain. The third line of defense uses tip selection algorithms to audit DAG branches, gradually degrading poorly performing branches and blocking malicious nodes from participating in global training, thereby enhancing DSFL’s robustness. DSFL enhances efficiency compared to conventional authentication techniques while effectively safeguarding privacy. The experimental results in Section 4.7 demonstrate that DSFL achieves robust performance on both balanced and imbalanced datasets under strict privacy-preserving conditions.

### 1.3. Fair Incentive Mechanism

In the past years, studies have discussed the incentive mechanisms of Blockchain-FL, focusing on encouraging more clients to participate in the FL. Nevertheless, few studies have addressed the issue of unfair incentives due to performance disparities. Differences in computational capabilities among clients may lead to unequal contributions to the federated model, which could indirectly result in unfair incentives, thereby affecting the learning process [9]. To promote the participation of diverse nodes and recognize the contributions of all well-intentioned nodes, we should enhance the fairness of incentives through algorithmic improvements.

Rehman et al. proposed a blockchain-based fine-grained reputation system using Ethereum’s public blockchain and smart contracts [10]. Toyoda et al. introduced a system that updates the competitive model for fair client compliance and increased revenue [11]. However, this relatively autonomous voting mechanism may lead to potential monopolies and collusion among clients seeking tokens. Additionally, clients with small sample sizes and insufficient information may have a low probability of spontaneously selecting high-performing models, especially in large-scale blockchain communities where individual clients might be misled by overhyped popular models. Moreover, the diversity of models can create challenges in reaching a consensus, as different nodes may select models best suited to their needs, resulting in high communication costs and resource consumption for the required voting process.

Gao et al. developed FGFL, a blockchain-based incentive governor for FL [12]. In most studies, nodes with lower computational power and higher latency may fail to receive incentives, as their model performance tends to fall below the average. Some studies have ranked the models, with those nodes performing below average treated as malicious, resulting in their exclusion from global aggregation and the loss of incentives [13]. This situation can lead to financial losses, as clients may invest resources without receiving adequate rewards. Additionally, clients with small sample sizes and insufficient information may have a low probability of selecting high-performing models. This issue is particularly pronounced in large-scale blockchain communities, where over-hyped popular models may mislead individual clients. Moreover, the diversity of models can create challenges in reaching a consensus, as different nodes may select models best suited to their needs, resulting in high communication costs and resource consumption for the voting process.

### 1.4. Our Approach

Based on the three aforementioned issues, we develop our framework. First, to address the concurrency limitation, we replace the traditional P2P model of single-blockchain with a P2PS DAG network topology. We retain some elements of traditional blockchain, such as hash encryption, smart contracts, transaction publishing, and on-chain communication, while improving the DAG consensus to replace the blockchain consensus. This modification enhances FL concurrency in a short time while ensuring client privacy and training decentralization, leading to greater efficiency.

Meanwhile, we propose utilizing a shard structure for synchronous training above the DAG layer, which helps achieve small-scale resource integration. By integrating the shard layer into the asynchronous DAG network, we can further enhance training efficiency, alleviate storage pressure on the DAG blockchain, and reduce the complexity of the DAG structure. By adjusting parameters, we can control the scale of our shard layer to regulate the pace of FL and the trajectory of the DAG blockchain, allowing our training tasks to progress toward the desired state and significantly speeding up large-scale training.

Regarding anti-interference, we retain the encryption mechanisms in the blockchain and propose DSFL, which enhances scalability and ensures the anti-interference capability of FL.

Finally, to establish a fair incentive mechanism that ensures even nodes with small data volumes and limited computational power receive fair rewards, we combined three random walk algorithms to develop our adaptive algorithm. We promote a more balanced DAG community, leading to a fairer incentive mechanism and encouraging greater client participation.

The contributions of this article are as follows:

1. We propose a DSFL framework that integrates the DAG-Shard-Publishers three-party system for FL tasks. Through a two-layer structural system and three audit protection lines, it achieves high concurrency, anti-interference, and a fair incentive mechanism.
2. We design three tip selection algorithms based on the DAG consensus and develop an adaptive algorithm with phased adjustments to ensure efficient training while achieving concurrent control of DAG.
3. We conduct simulation experiments to investigate the performance of DSFL in terms of sharding dimensions, algorithm dimensions, and parameter dimensions while also validating the advantages of high concurrency, fairness, and robustness across multiple datasets and commission mechanisms.

## 2. Related Work

This section provides a comprehensive review of prior research related to DSFL. Table 1 presents the main related work. We organize our discussion around four key dimensions to highlight various approaches and findings within the field.

### 2.1. Decentralized Federated Learning vs. Centralized Learning

On the one hand, traditional Centralized Learning (CL) requires transmitting complete data samples to a central server, which has a huge communication overhead [15]. On the other hand, CL poses challenges when dealing with sensitive data that clients are unwilling to disclose. Meanwhile, there is a risk of data leakage during transmission, making it difficult to collect sufficient data. Additionally, an attack on the central node in CL can lead to a single point of failure.

Therefore, Google proposed the first and most impactful federated learning system [16]. This system allows clients to train locally while storing their data on their devices, only uploading model weights and reducing the risk of data leakage. Furthermore, the distributed architecture disperses storage pressure, which enhances system fairness and fault tolerance [17]. This trend toward decentralization and democratization in the network ecosystem grants clients more autonomy in their choices.

### 2.2. DAG Blockchain vs. Blockchain

Blockchain is a commonly used privacy-preserving architecture in FL, and in recent years, the DAG blockchain has also gradually been applied in FL. Despite the characteristics of blockchain being public, transparent, and immutable, the speed and scalability of blockchain continue to pose challenges. The single-chain structure has concurrency limitations, making it unable to meet the low-latency requirements for model update confirmations [18]. To address the above issues, the Internet of Things Application (IOTA) ledger based on DAG is proposed, which processes many transactions more quickly by relying on a consensus scheme based on cumulative weights [19]. On the DAG blockchain, a new tip can select 2–8 parent tips, significantly improving the asynchronous concurrency of large-scale tasks quickly. The acyclic characteristic of DAG effectively avoids circular dependencies, ensuring independent concurrent updates across various devices, enhancing scalability and reducing latency.

Although DAG serves as a distributed ledger or cryptocurrency that can potentially replace blockchain [20], effectively reducing mining costs associated with blockchain, DAG still has some areas to improve. In Section 2.3, we introduce the advantages and disadvantages of DAG consensus, and in Section 2.4, we discuss the strengths and weaknesses of DAG-FL.

### 2.3. DAG Consensus vs. Blockchain Consensus

Using Proof of Work (PoW) in traditional Blockchain-FL leads to extensive calculations, making the verification process slow and inefficient. Blockchain relies on the mining process of PoW to control the single-chain model and prevent double-spending, which leads to significant time costs. In PoS blockchain consensus, more powerful devices, such as those in the IoT, serve as miners and earn the highest rewards for maintaining the ledger. This creates a situation where nodes with lower computational power struggle to receive rewards.

DAG consensus relies primarily on tip selection for node validation, offering an alternative to traditional blockchain mining. In conventional tip selection algorithms, the procedure takes a lot of time and resources. Some research has been conducted to improve tip selection algorithms. For instance, Hybrid Tip Selection (HTS) aims to identify relevant and irrelevant tips from non-IID data to prevent model overfitting [14]. The Reputation-based Tips Selection Algorithm (RTSA) is proposed to reduce blockchain consensus delays [21]. Gai et al. proposed a consensus that orders DAGs and merges them into a single chain [22]. Morais et al. introduced the Nero algorithm into DAG [23].

Another challenge with traditional DAG consensus is the “lazy” node. For example, a “lazy” node can continuously approve the oldest transactions and not contribute to approving recent transactions but still has an incentive. Furthermore, malicious entities can artificially inflate the number of tips by creating many transactions that approve specific fixed transactions [20]. Chen et al. proposed TDTS, a tip-selection algorithm based on time division, to improve the efficiency of transaction verification and avoid “lazy” and malicious nodes [24].

In response to the above-mentioned challenges, the Tip selection mechanism in DSFL integrates three random walk algorithms and accuracy-related DAG’s branch degradation to effectively prevent the presence of “lazy” nodes. Additionally, the random selection of these three walk algorithms through smart contracts helps mitigate the risk of malicious entities manipulating DAG nodes. Since smart contracts supervise our Tip selection algorithm, it ensures greater transparency, openness, and objectivity.

### 2.4. DSFL vs. DAG-FL

There are many existing studies about DAG-FL. Yuan et al. proposed a hierarchical Chains-FL architecture based on DAG and Raft consensus [2]. However, it fails to address concurrency and time efficiency in federated learning within DAG structures. Moreover, the impact of DAG network topology, DAG consensus, and shard consensus on overall training remains unexplored. Tan et al. proposed Personalized Federated Learning (PFL), which distinguishes between global models and locally personalized models [25]. However, the partitioning of data characteristics and sample space similarity can increase the risk of data leakage and collusion among malicious nodes. Wang et al. proposed that DAG-FL is more efficient than traditional Blockchain-FL [20]. However, the direct interaction between numerous clients and the DAG chain is less efficient than the hierarchical training process of our DSFL. Furthermore, the absence of a strict audit mechanism makes training time efficiency heavily dependent on the quality of models in the DAG. Additionally, relying solely on the α parameter to control the randomness or weight dependence in the random walk algorithm without a DAG committee to ensure real-time node accuracy can lead to over-reliance on historical weights. This limits the ability to assess current training performance and makes the system vulnerable to single-point attacks that alter a node’s weight, causing the DAG to drift toward a specific branch. The integrated algorithm in DSFL addresses these issues and reduces reliance on single weights during random walks.

In DAG-FL, another discussion is about synchronization and asynchronization. Wang et al. proposed a mechanism that clients interact asynchronously with DAG [14]. However, relying solely on asynchronous aggregation can degrade the performance of DAG, as clients typically have a limited number of data samples. This situation makes it easy for the aggregation to incorporate outdated models from earlier times, leading to a decline in model performance and training efficiency. Moreover, as the number of clients increases, the scale of DAG becomes unmanageable. This results in reduced efficiency of the random walk algorithms and places excessive storage pressure on the DAG blockchain. Ko et al. further introduced the concepts of sub-DAGs for edge devices and AFL-DAG [26]. Tangle uses a DAG data structure to store the transactions, allowing multiple transactions to be added to the ledger simultaneously, and it can achieve consensus similar to the Nakamoto DLT [27].

The current research on DAG-FL rarely addresses how DAG topology, DAG consensus, and shard consensus affect FL training. Our DSFL approach combines synchronous and asynchronous methods: shard-level synchronous aggregation improves short-term accuracy, while mainchain asynchrony ensures coherence across shards. The two-layer architecture allows DAG scaling by adjusting shard size, with raft-shard or commission shard consensus chosen based on shard size.

## 3. Framework

**Figure 1:**
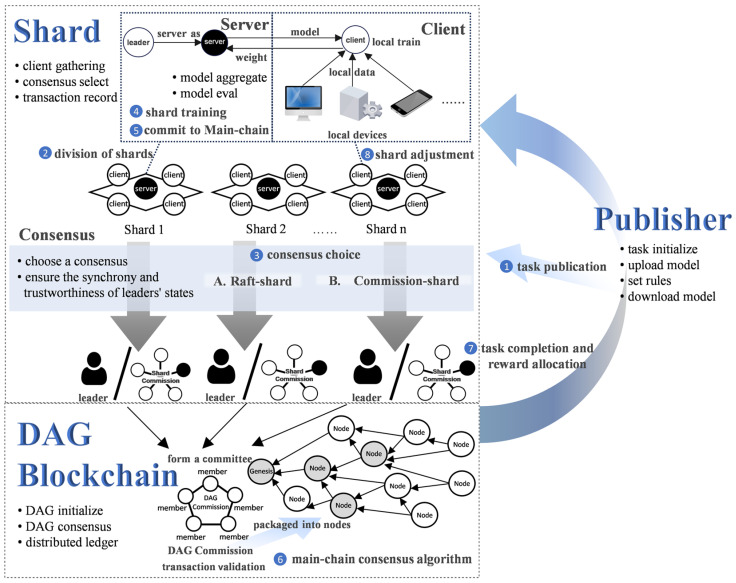
DSFL architecture.

In this section, we illustrate the framework and workflow of DSFL. As shown in Figure 1, DSFL comprises three components: publisher module, shard module, and DAG blockchain module, along with eight phases: ① task publication, ② division of shards, ③ consensus choice and review system mechanism, ④ shard training, ⑤ commit to main chain, ⑥ main chain consensus algorithm, ⑦ task completion and reward allocation, ⑧ shard adjustment. The Publisher module focuses on stages ① and ⑦, the shard module is responsible for stages ②, ③, ④, ⑤, and ⑧, while the DAG chain module primarily manages stages ⑥ and ⑦.

### 3.1. Publisher Module

In phase ①, the publisher submits the initial model weights and the expected reward distribution ratio to the smart contract on DAG. After this submission, the execution of the tasks is entirely managed by the smart contract. The task publisher cannot stop or intervene in the FL tasks during training. This way, we can make the system more transparent and avoid the situation where publishers collude with specific nodes to gain unfair incentives. The publisher takes the well-trained model from the DAG blockchain in phase ⑧.

### 3.2. Shard Module

The shard comprises three components, server, client, and consensus, and is responsible for synchronized federated learning.

- The Server is served by the leader or a committee selected through different consensuses. It handles shard-level FL aggregation, clients’ weight verification, and transaction submission to DAG.
- The clients, comprising the nodes within the shard, are responsible for local training in FL. To ensure data privacy, raw data are not uploaded. Clients transmit only the model weights to the server.
- The consensus is to ensure that distributed learning in the shard is fair and works properly. The consensus can either be a raft–shard consensus or a commission–shard consensus.

Once the consensus mechanism is selected, each shard primarily adopts the FedAvg algorithm in FL, which is robust for non-iid data and reduces communication rounds needed for deep network training on decentralized data.

**Figure 2:**
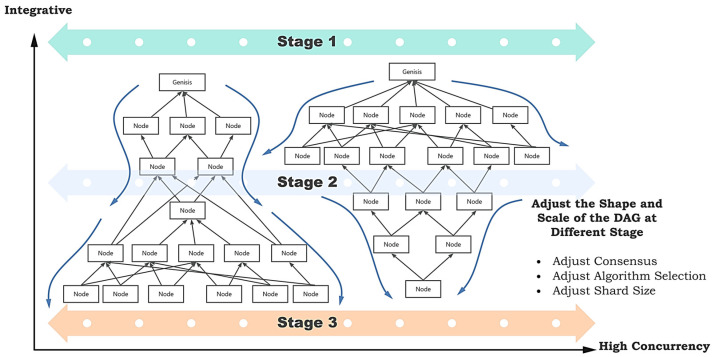
Concurrency control and phased adjustment of DAG morphology (Stages 1–3 represent the first, middle and last stages of DSFL. The arrows indicate changes in the size of the DAG).

Concurrency control is one of the central topics addressed in this paper. As shown in Figure 2, we divide the entire training flow into multiple periods. In each period, we control the concurrency of DAG by adjusting the shard size, the shard consensus algorithm, and the ratio of three tip selection algorithms. By making adjustments, we can achieve an initial divergence followed by convergence or initial convergence followed by divergence to alter training efficiency.

**Figure 3:**
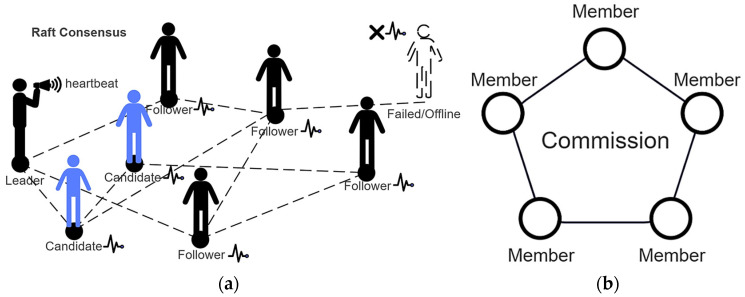
Two types of consensus algorithms within the shard. (**a**) Raft–shard consensus; (**b**) Commission–shard consensus.

Another essential component within the shard is the two consensus mechanisms shown as Figure 3. The specific details are as follows as we redesign the core concepts of the consensus.

#### 3.2.1. Raft–Shard Consensus Mechanism

Raft Consensus is a distributed log consensus suited for the leadership selection in the small size of the shard. We improved the raft functions to better fit FL in the shard. The participants are divided into three roles: leader, follower, and candidate. The leader collects the weights uploaded by clients and completes the aggregation, and the model parameters are then broadcasted to other nodes. The followers receive and store the model parameters published by the leader and perform local training. Candidates are responsible for assuming the leader’s tasks if the leader fails. The specific process is as follows.

Client $C_{ij}$ in the shard will first form a node $N\left(s_{i},c_{ij},w\right)$ along with shard information $s_{i}$, client reputation value w, and client information. At the initial moment, all nodes are in the follower state. Each node must receive a heartbeat from the leader within its election timeout to ensure the leader exists and functions appropriately. If a follower does not receive a timely heartbeat, it will proactively initiate an election and change its role to candidate. The term, denoted as Term, is a crucial concept in Raft consensus, serving as a timing mechanism to ensure the real-time and effective operation of consensus. After participating in the election, each candidate first votes for themself and then sends vote requests to the followers, waiting for responses from other nodes.

When a follower receives a request, if the requested ${Term}_{r}$ is greater than its current ${Term}_{C}$, or if it is in the same term but has not yet voted, the follower will vote for this candidate. Additionally, if the candidate’s ${Term}_{r}$ is greater than the follower’s ${Term}_{C}$, the follower will update its term to the new term. This is illustrated in Equation (1):

(1) $${\;Term}_{r}>{Term}_{C}\to {Term}_{r}={Term}_{C}.$$

The mechanism for determining whether a voter casts a vote for a candidate is designed as follows:

- In any term, within a single-leader structure in a shard, each node can cast only one vote. If there are many clients in the shard, multiple leaders can be elected.
- The information known by the candidate cannot be less than their own to ensure the freshness of *Term*.

Three possible outcomes may occur after the voting concludes:

1. Receiving votes from more than half of the participants, thus winning the election and becoming the leader.
2. Being informed that someone else has been elected, resulting in a switch to the follower state.
3. Not receiving the majority of votes within a certain time frame and not being notified of another candidate’s election, thus remaining in the candidate state and initiating a new election.

Each candidate checks their vote count, and if more than half of the nodes have voted for that candidate, they become the leader. Once the node becomes the leader, it must continuously send heartbeat messages to the other nodes to ensure it can effectively manage the shard and inform them of its status. Upon receiving the heartbeat, the other nodes will reset their countdown timers. If the countdown expires without receiving another heartbeat, it indicates that the previous leader may encounter an issue, and a new election will be prompted among the candidates.

To maintain information consistency and traceability within the shard, we implement a logging mechanism that records key details such as the current leader’s term number and client command requests. The log for node i is denoted as $L_{i}$, where $I_{prev}$ represents the index of the previous log entry, and entry refers to the log entry that is to be appended.

When the leader receives a request from a client, if ${Term}_{r}<{Term}_{i}$, the request is ignored. If the request is accepted, it is written to the leader’s pre-commit log and then broadcasted to other nodes. If other nodes find a match in their logs, they update their state to follower, reset their voting targets, and check the log matching conditions. If $I_{prev}$ is invalid or does not match in the current L, the log is truncated, retaining entries only up to $I_{prev}$ as (2):

(2) $$I_{Prev}\geq \left|L_{i}\right|\;or\;L_{i}\left[I_{Prev}\right]\neq entry\left[0\right]\;\Rightarrow \;L_{i}=L_{i}\left[:I_{prev}\right].$$

After a node receives a synchronization request, it will compare its log with the leader’s previous log. If there are any missing log entries, the node will use the corresponding entries from the leader’s log to fill in the gaps and then append the new log entries to its own log as (3):

(3) $$entry\neq None\wedge \left(I_{Prev}\geq \left|L_{i}\right|\;or\;L_{i}\left[I_{Prev}\right]\neq entry\left[0\right]\right)\Rightarrow L_{i}\leftarrow L_{i}\cup entry.$$

If more than half of the nodes in the shard, including the leader itself, provide positive responses to the leader, the leader will add the request to the pre-commit log. At this point, the leader will “commit” the request, respond to the client indicating that the write request has been successfully processed, and update the commit index for each client.

**Figure 4:**
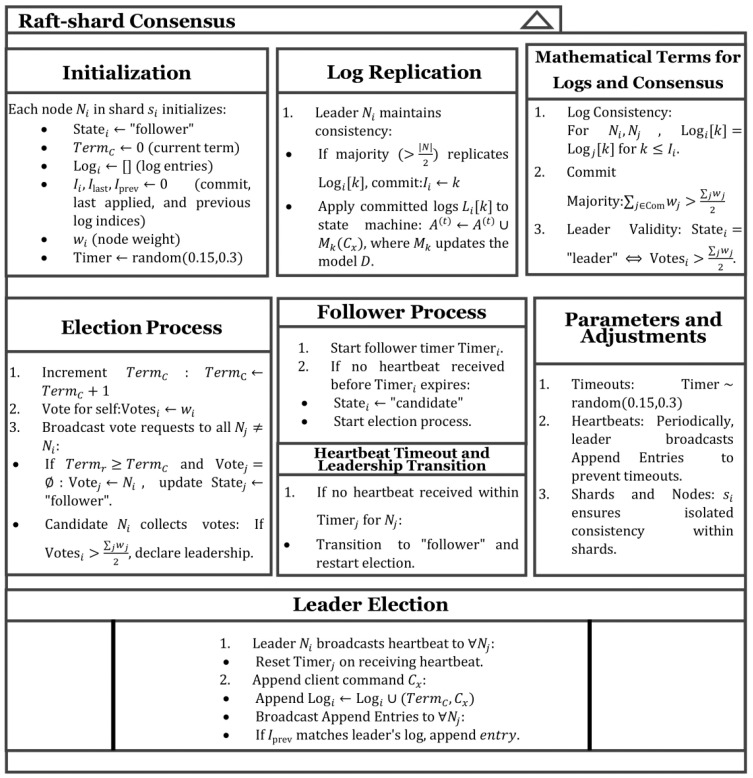
Pseudo-code class diagram for raft–shard consensus.

In the subsequent period, other nodes will become aware of this “commit” action through interactions with the leader. They will also commit this request in their pre-commit logs, which qualifies the committed pre-commit log entries to be applied to the state machine. $I_{i}$ represents the index that node i has committed, and $I_{last}$ represents the last index applied to the state machine by node i. For each un-applied log entry j that satisfies $I_{i}<j\leq I_{last}$, it will be applied to the state machine, and the application index will be updated accordingly. Then, the leader will aggregate the weights submitted by all nodes within the shard. The pseudo-code class diagram for the entire consensus is shown in Figure 4.

#### 3.2.2. Commission–Shard Consensus Mechanism

In cases where there are many participants within the shard, a committee consensus is more suitable. Committee consensus allows for collective decision-making by involving multiple participants, which helps to diversify opinions and distribute risks. With a larger committee, the necessity for continuous heartbeat synchronization to maintain the real-time status of decision-makers is eliminated, thereby conserving communication resources and enhancing operational efficiency. However, a multi-person committee’s decision-making process is generally slower than the single-leader decision-making of the raft consensus.

$N_{i}$ represents the i-th node, and Com denotes the committee members. The selection of committee members is based on the historical accumulated weights, which reflects each node’s performance. The weight is determined by the node’s reputation and credibility, serving as an important criterion for electing the committee. The nodes are arranged in descending order according to their weights $w_{i}$, and the top k nodes with the highest weights selected to form the committee Com:

(4) $$Com=\left\{\left.N_{i}\right|w_{i}\geq w_{j},j=1\cdot \cdots k\right\}.$$

For each member $N_{i}$ in the shard, the committee uses the local models of the committee members and the training data $D_{train}$ to evaluate the accuracy of the models of all nodes except itself. The committee assesses the model ${acc}_{ij}$ of $N_{i}$ for model $M_{j}$ as (5):

(5) $${acc}_{ij}=\frac{\Sigma_{x\epsilon {D_{t}}^{’}}1\left(argmaxMi\left(x\right)=y\left(x\right)\right)}{\left|{D_{train}}^{’}\right|},$$

here, 1(⋅) is the indicator function that determines whether the prediction is correct, y(x) is the true label of sample x, and ${D_{train}}^{’}$ is a subset drawn from the global training set $D_{train}$.

Committee calculates the final score ${sco}_{i}$ for the node $N_{i}$ as (6):

(6) $${sco}_{i}=\frac{1}{\left|Com\right|}\sum_{N_{i}\in Com,i\neq j}{acc}_{ij}.$$

**Figure 5:**
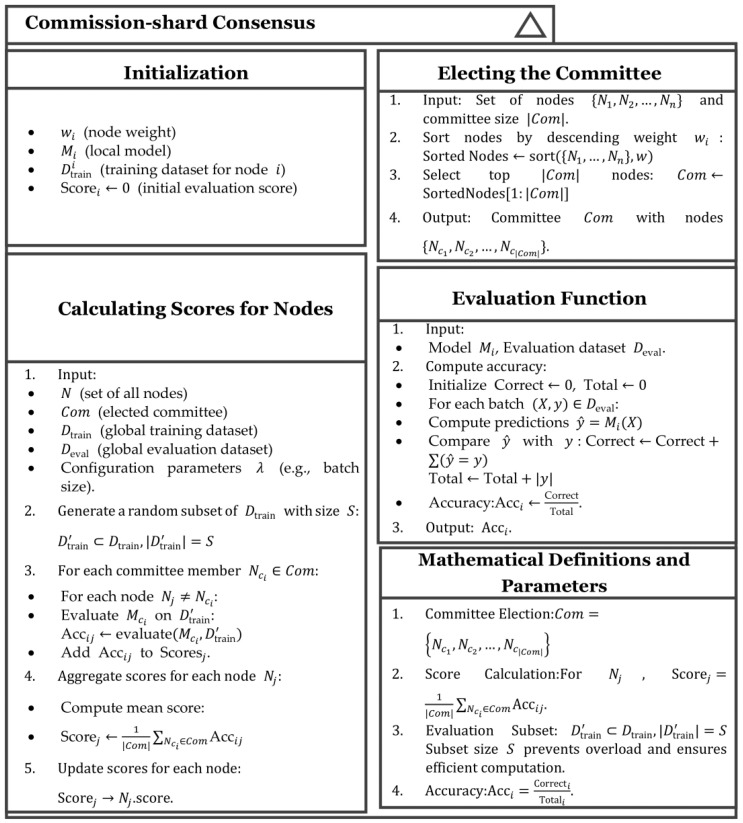
Pseudo-code class diagram for commission–shard consensus.

Based on the accuracy scores, the shard committee selects the top several nodes with the highest scores from each shard for model aggregation. The pseudo-code class diagram for the entire consensus is shown in Figure 5.

### 3.3. DAG Chain Module

The DAG blockchain is used for asynchronous FL, audit traceability, random walk branch aggregation, and incentive distribution. In phase ⑥, DAG completes the global model aggregation through tip selection and distributes incentives to all shards in phase ⑦.

#### 3.3.1. Function and Structure of DAG

The transaction storage structure of DAG consists of four components. As shown in Figure 2, all nodes in DAG represent transactions. The first transaction pools all transaction records on DAG, and the approved transaction pool contains validated transactions. The tips pool consists of unvalidated transactions.

**Figure 6:**
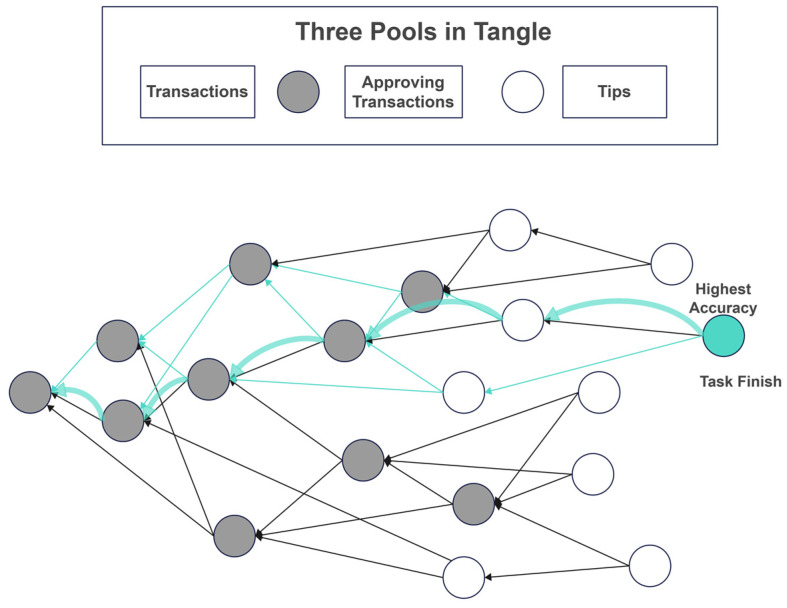
DAG storage system and topology (The green arrow represents the main path of backtracking).

The DAG serves as a tool for asynchronous shard-level FL aggregation. By leveraging predecessor pointers, the DAG can verify partially validated tips already added to the DAG and perform localized FL aggregation tasks. This approach avoids the inefficiencies of full validation required for global aggregation. As shown in Figure 6, nodes are added layer by layer, directly or indirectly aggregating and verifying preceding transactions. An asynchronous setup eliminates the need for synchronization across all shard states, better accommodating large-scale concurrent asynchronous scenarios. Additionally, it mitigates the efficiency loss in synchronous aggregation caused by variations in the training capabilities of different nodes. We utilize hash functions on the DAG blockchain to generate unique hash values that link blocks and transactions. Each block in DAG is linked to the previous m blocks as parents through hash pointers, forming a mesh structure that guarantees the immutability of each block once it is created. m is generally 2–8, but having two parents often results in better validation efficiency. While having more than two parents can provide a more comprehensive model aggregation, too many parents decrease time efficiency, which is not conducive to the rapid expansion of DAG. Therefore, two parent nodes are often used, as the layer-by-layer validation of the tip also indirectly validates the ancestor nodes of its parents. Once a tip is validated by k approvers, it moves from the tip pool to the approved transactions pool. The value of m significantly affects the load characteristics of DAG. The adjustments of m to achieve either low load or high load conditions are extensively discussed in [15,20], so they will not be a primary focus of this paper.

The main functions of DAG include creating the tangle objects, initializing the transaction dictionary, setting up the Genesis, calculating the depth of all transactions, initializing the server and model, and adding weights to transactions along the random walk path. Depth is defined as the length of the longest directed path from a tip t to the transaction Txn by using Equation (7):

(7) $$depth\left(Txn\right)=\underset{t\in tips}{max}\;\mathrm{length}\left(path\left(t,Txn\right)\right),$$

we can retrieve all transaction IDs within a specified depth range from the depth cache, enabling limited-range retrieval within DAG.

After the training task reaches the threshold for completion, we introduce the concept of multi-edge, which enhances the functionality of the DAG structure and allows for the identification of main branches within complex network architectures.

As shown in Figure 6, after the training task concludes, the publisher retrieves the model weights with the highest accuracy from DAG and traces the walking path, defining this path as the main path of the DAG blockchain. We can establish multi-edge on the main path for the following reasons.

- Authority in temporal ranking: In a large-scale concurrent DAG structure, we use the transaction order of the main path as the main chain’s temporal standard to prevent double-spending issues.
- Higher Weight: In subsequent task publishing processes, the main path carries a higher weight, increasing its likelihood of being selected in the tip selection algorithm.
- Rational Reward Distribution: We can assess the contribution of other participating task nodes based on the main path, which allows for a fair calculation of the token distribution among other shards.

Knowing the general direction of the main path enables the strategic allocation of storage, computational, and communication resources along this trajectory. The main path is similar to a traditional blockchain structure. DSFL preserves the PFL training results for each shard along the sub-paths while helping the model on the main path converge faster, saving both time and space.

#### 3.3.2. DAG Consensus

The main DAG consensus involves three tip selection algorithms to find parent nodes while the parent nodes find their approvers, who evaluate and then add them to the main chain’s tip pool. Based on the DAG–shard structure, we design three types of tip selection algorithms and control the selection ratio of the algorithms by $\lambda_{1}$ and $\lambda_{2}$. Furthermore, the entire training process can be divided into different phases, allowing the algorithm ratio to be adjusted by varying $\lambda_{1}$ and $\lambda_{2}$ in each phase, thereby enhancing the expansion speed of DAG, its growth morphology, and the training efficiency of tasks.

In previous studies, random walk algorithms have primarily relied solely on weights, which carries certain risks. Therefore, we consider other factors like fairness. Hence, the three tip selection algorithms of DSFL serve different functions: Algorithm 1 focuses on horizontal concurrency walking, Algorithm 2 emphasizes rapid experiential walking based on accumulated experience and established information, and Algorithm 3 concentrates on vertical extension walking. By integrating these three algorithms, we can use $\lambda_{1}$ and $\lambda_{2}$ to comprehensively regulate the scale of DAG, short-term efficient scalability, the fairness of the DAG community, and training efficiency. Let us introduce the specific implementation principles of these algorithms.
**Algorithm 1** Random Walk Tip Selection  1: **Input:** Genesis node *G*, Tangle *T*, Trace set T_trace_, Maximum steps *S*_max_  2: **Output:** Selected tip node   3: current node ←*G*  4: T_trace_ ←{current node.id}  5: steps ← 0  6: **while** steps *<S*_max_
**do**  7:  **if** current node ∈T_tips_
**then**  8:   **break**  9:  **end if**10:  approvers list ←A(current node)11:  **if** approvers list = ∅ **then**12:   current node ←*G*13:   T_trace_ ←∅14:   **continue**15:  **end if**16:  current node ← rand(approvers list)17:  T_trace_ ←T_trace_ ∪ {current node.id}18:  steps ← steps + 119: **end while**20: **if** steps ≥*S*_max_
**then**21:  current node ← rand(T_tips_)22: **end if**23: **return** current node

Algorithm 1: The random walk in Algorithm 1 stops upon encountering the first tip, which enhances the horizontal concurrency of DAG. The algorithm performs p rounds of wandering, after which it evaluates the found tips’ performances based on the global test set. In each round of wandering, two tips are selected and evaluated. After p rounds, the highest accuracy set of tips is recorded as its parent nodes, and it joins the approvers of these two parents. The specific process of the algorithm is shown in Algorithm 1, where G is the Genesis node, T is the main chain of the tangle, $t_{i}$ represents the i-th tip, A(n) is the set of approvers for node n, T is the trace set, $S_{max}$ represents the maximum number of steps to prevent wandering into a deadlock, rand(X) denotes randomly selecting an element from set X, while $t_{1},t_{2}$ represent the selected two tips. If the algorithm falls into a deadlock, a tip will be chosen from the tip pool on DAG.

Algorithm 1 requires more evaluation time as the number of rounds p increases. While more rounds improve the chances of finding a better-performing model, it is crucial to balance p between time constraints and performance goals.
**Algorithm 2** Two-Way Random Walk for Tip Selection  1: **Input:** Genesis node *n*_1_, Tangle *T* = ⟨*N*,*E*⟩, Maximum iterations *S*_max_  2: **Output:** Two selected tips *n*_1_,*n*_2_ and their traces T_trace1_,T_trace2_  3: *n*_1_ ← *n*_1_ (initialize wandering path 1)  4: *n*_2_ ← *n*_1_ (initialize wandering path 2)  5: Ttrace1 ← {*n*1*.id*}  6: Ttrace2 ← {*n*2*.id*}  7: visited1,visited2 ← ∅  8: **function** update current node(current node)  9:  A(*n*) ← {*a_i_* ∈ T | *a_i_* ∈ approvers of *n*}10:  max_1_,max_2_ ← −∞,−∞ (initialize maximum weights)11:  *n*_1_,*n*_2_ ← ∅,∅ (initialize next nodes)12:  **for** each *a_i_* ∈ A(*n*) **do**13:   **if** weight*_ai_ >* max_1_
**then**14:     max_2_ ← max_1_,   *n*_2_ ← *n*_1_15:     max_1_ ← weight(*a_i_*), *n*_1_ ← *a_i_*16:   **else if** weight*_ai_ >* max_2_
**then**17:     max_2_ ← weight(*a_i_*),   *n*_2_ ← *a_i_*18:   **end if**19:  **end for**20:  **return**
*n*_1_,*n*_2_21: **end function**22: While A(n_1_) ≠ ∅ and iteration count *< S*_max_23:  visited1 ← visited1 ∪ {*n*1*.id*}24:  *n*_1_ ←update current node(*n*_1_)25:  Ttrace1 ← Ttrace1 ∪ {*n*1*.id*}26:  **if** iteration count ≥ *S*_max_
**then**27:   *n*_1_ ← rand(T_tips_visited1)28:  **end if**29:  Reset iteration count30: **While** A(n_2_) ≠ ∅ and iteration count *< S*_max_31:  visited2 ← visited2 ∪ {*n*2*.id*}32:  *n*_2_ ←update current node(*n*_2_)33:  Ttrace2 ← Ttrace2 ∪ {*n*2*.id*}34:  **if** iteration count ≥ *S*_max_
**then**35:   *n*_2_ ← rand(T_tips_ \visited2\{*n*_1_*.id*})36:  **end if**37: **Return**
*n*1, *n*2, Ttrace1, Ttrace2

Algorithm 2 primarily conducts random walks based on established facts (weights). Since it does not require evaluation, the walking time is relatively quick. However, solely relying on weights for the random walk can easily perpetuate advantages for a particular branch. If a model trained by a certain node performs well initially, following the weight-based approach will create a snowball effect, further amplifying its advantage and leading to unfairness for other nodes. Additionally, a model that performs well initially does not necessarily guarantee continued good performance in subsequent training, so the proportion of Algorithm 2 must be considered.

The details of Algorithm 2 are introduced above: $V_{i}$ denotes the set of transactions accessed by the i-th trajectory, and max_nodes(A) returns the two transactions in set A with the highest and second-highest weights, respectively. The algorithm aims to find the two tips with the highest weights by choosing nodes with larger weights to wander. If the algorithm reaches the maximum number of iterations without finding suitable tips or if it falls into a deadlock, it will randomly select a tip from the tip pool of the main chain.
**Algorithm 3** Random Walk Select Tip**Require:** Start node *G*, Tangle *T*, Trace set T, Maximum steps *S*_max_  1: *n* ← *G*  2: Add *n.id* to T
  3: Initialize *V* ←∅        ▷ Visited nodes  4: *k* ← 0  5: **while**
*n*.approver ≠∅ and *k < S*_max_
**do**  6:  approvers list ← list of *n*.approver.values()  7:  **if** approvers list is empty **then**  8:   *k* ← *k* + 1  9:   *n* ← *G*10:   Clear T
11:  **end if**12:  *n* ← Random choice from approvers list13:  Add *n.id* to T
14:  *k* ← *k* + 115: **end while**16: **if** *k* ≥ *S*_max_
**then**17:  tips list ← list of *T*.tips.values()18:  **if** tips list is empty **then**19:   Raise ValueError(”No tips available to choose from.”)20:  **end if**21:  *n* ← Random choice from tips list22: **end if**23: **return** *n*

Algorithm 3: The wandering algorithm stops when a node has no approvers, as shown above. This algorithm offers a better vertical extension for DAG. The specific process is similar to that of Algorithm 1, as it finds the tips with the highest accuracy after p rounds. Overall, the execution time is slightly longer than Algorithms 1 and 2. Through the control of Algorithms 1 and 3, the overall trend of DAG can be adjusted.

Finally, two tips and their traces are identified for the highest accuracy. Once the tips are recognized as the node’s parents, the node is added to the tips pool of the DAG blockchain.

### 3.4. Entire Workflow

We implement synchronization within shards and asynchronous implementation on the DAG blockchain. Meanwhile, DSFL features a two-layer structural system and three lines of defense. The first line of defense involves an initial review by the internal auditing committee of each shard. We propose two committee consensuses tailored for large and small shards, allowing for the fair and reasonable election of shard leaders. Shard leaders can identify useless models and potential malicious nodes within the shard, preventing bad models from polluting the DAG blockchain and wasting resources before transactions are recorded. The second line of defense is the DAG committee, composed of the shard leaders, which effectively balances the development of the global model and prevents potentially attacked shard models from being submitted on-chain. The third line of defense involves auditing tips using different tip selection algorithms. Branches on DAG with poorly performing parameters will gradually degrade, effectively blocking malicious nodes from participating in subsequent global model training, thereby enhancing the anti-interference capability of DSFL. The eight phases cycle repeatedly between tasks. The main process is as follows.

#### 3.4.1. Phase 1: Task Publication

We establish a review mechanism during the task publishing phase to prevent malicious task publishers from monopolizing DAG resources. The task publisher submits the relevant weights, hyperparameters, thresholds, and incentive mechanisms to the smart contract. The smart contract reviews the feasibility and resource consumption of the task within a limited time frame. Upon approval, it initializes the Genesis block. It publicizes the task, allowing clients who respond within the specified time to join the client list $C=\left\{c_{1},c_{2}\cdot \cdots c_{n}\right\}$, where each client possesses a local dataset $D=\left\{d_{1},d_{2}\cdot \cdots d_{n}\right\}$. Clients who do not respond within the given time are considered to have forfeited the task.

#### 3.4.2. Phase 2: Division of Shards

Managing a large client base in decentralized FL is crucial for efficiency, helping to avoid issues like excessive DAG size, high resource consumption, and slow aggregation convergence from direct client interactions. We use Shards $S=\left\{s_{1},s_{2}\cdot \cdots s_{m}\right\}$ for hierarchical management of the large client population and initial aggregation of models within each shard. If there are too many shards, they may fail to provide effective management and constraints. Conversely, having too few shards in a large client group makes it challenging to ensure that all clients within a shard can synchronize, leading to potential centralization risks.

In terms of shard division, if shards are clustered based on the similarity of sample features in client datasets, the division of shard groups may still reveal approximate data characteristics of other clients. This could happen even if clients do not upload sensitive data during local training. While this approach can enhance the learning efficiency of PFL groups, it poses a leakage risk in privacy-sensitive fields such as healthcare and finance, making FL client-unfriendly. Therefore, we aim to simulate a more realistic scenario, where clients are randomly assigned to shards, ensuring that the sizes of the shards remain as equal as possible. Each shard will consist of k clients, represented as $s_{j}=\left\{c_{i1},c_{i2}\cdot \cdots \cdots c_{ik}\right\},j=1,2\cdot \cdots m$. This approach actually establishes the proportion coefficient $\frac{k}{n}$ for server nodes within the entire FL network.

#### 3.4.3. Phase 3: Consensus Choice and Review System Establishment

To select organizations that represent shards for aggregating models and auditing models on-chain, we can adapt the consensus based on the size of the shard. Raft is well-suited for small cluster environments that require strict data consistency, as it allows for flexible leader replacement. However, when the number of members in shard increases, leader broadcasting can consume significant communication resources, making committee consensus a more appropriate approach. Additionally, different consensus algorithms can be chosen based on the process stages and task volume of the algorithm.

#### 3.4.4. Phase 4: Shard Training

Based on the consensus, the leader or commission selected among clients serves as the shard server responsible for model aggregation and uploading. Each client joins a shard as a node N = $\left\{C_{1},C_{2\cdot }\cdot \cdots C_{n}\right\}$. Before conducting FL within the shard, there will be intra-shard broadcast communication to select real-time online clients for training aggregation. We use the FedAvg algorithm. The leader distributes the global model M to all clients. Clients train on their local datasets $D_{i}$ and upload their accumulated gradients.

The weights of the global model at the t-th iteration are denoted as $w^{\left(t\right)}$. $C_{i}$ performs local training based on the global model:

(8) $$\Delta w_{i}^{\left(t\right)}={argmin}_{\Delta w_{i}}f_{i}\left(w^{\left(t\right)}+\Delta w_{i}\right),$$

and updates weights $\Delta w_{i}^{\left(t\right)}$. The server aggregates the weights from all clients into a total accumulator $A^{\left(t\right)}$ and then computes the weighted average of the updates from all clients as (9):

(9) $$A^{\left(t\right)}=\sum_{i=1}^{N}\frac{D_{i}}{D}\cdot \Delta w_{i}^{\left(t\right)}.$$

In the case of raft consensus, the leader aggregates the gradients from all participating training nodes. In the case of committee consensus, only the gradients from the selected nodes with the highest scores are aggregated. The server updates the global model weights using $A^{\left(t\right)}$, and η is the learning rate:

(10) $$w^{\left(t+1\right)}=w^{\left(t\right)}+\eta \cdot A^{\left(t\right)}.$$

The shard training continues until the specified epoch is reached, at which point the training concludes. Training information is recorded in the log on the shard, and after the model is reviewed by the leader or committee, it is packaged into a transaction and sent to the DAG blockchain.

#### 3.4.5. Phase 5: Commit to Mainchain

The DAG committee comprises leaders from the shards, responsible for reviewing transactions from the shards. Transactions will join the DAG blockchain if the accuracy of the model exceeds a preset threshold. Reviewed transactions on DAG carry transaction information, shard information, and training performance such as accuracy, loss, recall, and F1 score, which will be added to the transactions pool of DAG as Algorithm 4.
**Algorithm 4** Commit Transactions to DAG**Require:***T*, *N*, Epoch *e*, acc, loss, recall, f1  1: Metadata(*T*, {issuer, timestamp, loss, acc, recall, f1})  2: DAG.transactions[*T*.id] ← *T*  3: **for** each parent *p_i_* ∈ *T*.parents **do**  4:  *p_i_*.approver[*T*.id] ← *T*  5:  **if** all *p_i_*.approved = True for all *p_i_* ∈ *T*.parents **then**  6:   *T*.approved ← True  7:  **else**  8:   *T*.approved ← False  9:  **end if**10: **end for**11: DAG.all transactions[*T*.id] ← *T*.parents12: Log(DAG.all transactions, DAG.approving transactions, DAG.tips)

#### 3.4.6. Phase 6: Main Chain Consensus Algorithm

After being reviewed by the DAG committee, transactions, through the DAG consensus algorithm, will identify tips to verify whether their accuracy exceeds a predefined threshold. If the verification passes, the tips will become the node’s parents, but the tips will be discarded if it fails. Suppose most subsequent nodes fail to verify on a certain tip and exceed the specified time limit. In this case, the tip will invalidate its training data and prevent the branch from continuing, which leads to degradation.

**Figure 7:**
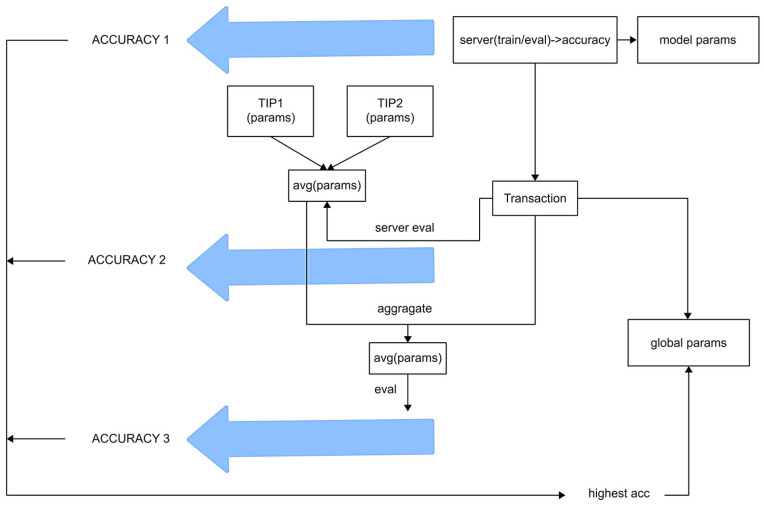
Principle of selecting optimal parameters.

After the integrated random walk algorithm using the three algorithms, let us assume that m tips have been verified through the audit process. The next step is model aggregation. The entire weight update process is illustrated in Figure 7. Through training, we can obtain the model ${acc}_{1}$ on the shard and $w_{local}$ of the shard. The total weight key set is $K=\left\{\left.k\right|k\in P_{1}\cup P_{2}\ldots \cup P_{m}\right\}$. Subsequently, weight alignment will be performed as shown in Equation (11):

(11) $$w_{i}\left(k\right)=\left\{\begin{array}{l} Tip_{i}\left[k\right]\;\;k\in {Tip}_{i}\\ 0\;\;\;\;\;\;k\notin {Tip}_{i} \end{array}\right..$$

After alignment, update the total weight dictionary as (12):

(12) $$W_{total}\left(k\right)=\sum_{i=1}^{m}w_{i}\left(k\right)\;\;\forall \;k\in K.$$

Then, average the weights according to the FedAvg algorithm as (13):

(13) $$W_{avg}\left(k\right)=\frac{1}{m}W_{total}\left(k\right)\;\;\forall \;k\in K.$$

Next, aggregate and evaluate $W_{avg}$ to obtain ${acc}_{2}$ and $w_{global}$. Then, merge $W_{avg}\left(k\right)$ with the weights of the model on the shard to get ${acc}_{3}$ and ${w_{global}}^{’}$. Compare ${acc}_{1}$,$\;{acc}_{2}$, and ${acc}_{3}$, recording the aggregation weights with the highest accuracy as the $w_{global}$ on the DAG blockchain. The transaction information keeps the highest accuracy and the local, personalized model training weights $w_{local}$ also recorded on the DAG blockchain. Finally, the shard leader distributes the new $w_{global}$ to the clients within the shard for the next round of training.

#### 3.4.7. Phase 7: Task Completion and Reward Allocation

Training ends when the specified number of iterations is reached or the task threshold is met. The publisher can retrieve the most optimal global model weights from the DAG blockchain based on the highest accuracy. They will trace back along the main path according to the node where the model resides, following the parent branches. Smart contracts or on-chain committees will distribute tokens to the shards based on the main path order, the weights of the DAG blockchain nodes, and their responsiveness. Within each shard, the leadership organization will distribute tokens to participating clients according to their contributions, which are primarily assessed based on model training accuracy, submission efficiency, and other relevant factors.

#### 3.4.8. Phase 8: Shard Adjustment

After each task round is completed, rewards are settled. Before the next round begins, clients can choose different shards based on their new data or make real-time adjustments to create new shards if the number of participants changes. The system can also automatically cluster clients into the same shard based on the branches of DAG, enhancing the cohesiveness within the shard and making the local, personalized models more targeted. Once the shards are formed, adjustments to the committees and leadership organizations are made within the shards and on the DAG blockchain.

Through the above eight phases, the entire FL task can be completed. Each DAG node stores both the local model weights and the global optimal parameters. Each node also functions as a distributed ledger, while the smart contracts on the DAG blockchain serve as contractual agreements, facilitating subsequent audits and traceability.

## 4. Experiments

In this section, we evaluate the performance of DSFL in terms of high concurrency, fairness, and robustness. We conducted seven experimental studies. Shard Dimension: (1) Impact of shard size on the performance of DSFL. (2) Effect of shard size on DAG growth patterns. Model and Algorithm Dimension: (3) Comparative experiments with different datasets. (4) Accuracy of the adaptive algorithm. (5) Impact of the adaptive algorithm on the fairness of the system. (6) Comparative experiments in class unbalanced scenarios. Model Parameter Dimension: (7) Performance experiments with datasets such as CIFAR-10, MNIST, and CIFAR-100, as well as models like ResNet18 and ResNet34, tested under different shard consensus mechanisms.

### 4.1. Experimental Setup

#### 4.1.1. Experimental Environment

Experiments use an x86, 64-bit architecture equipped with 30 GB of memory, an Intel Xeon Silver 4110 processor (16 cores, 32 threads) (Santa Clara, CA, USA) at a base clock of 2.10 GHz, and virtualization support, along with an L3 cache. For GPU-accelerated computations, the graphics driver version 515.57 supports CUDA 11.7. This setup offers efficient multi-core performance for compute-intensive tasks, with Python3.9.19-based simulations of DAG and shard frameworks.

To enhance model concurrency, we implement multithreading to simulate high-synchronization, large-scale data training. A lock-based threading approach prevents resource deadlocks, while a thread pool simulates the concurrent process. Although training runs on a single machine, dividing tasks into multiple time slices creates an effective serial workflow, allowing better simulation of real-world, large-scale concurrency scenarios.

Our goal is to explore the impact of shard size on FL. Currently, few studies have investigated this aspect. Identifying how shard size affects FL will provide valuable insights. To simulate the concurrent training of multiple shards on a single machine, we first need to eliminate the time delay caused by concurrent shard training on one machine. This is because there is less delay when multiple shards are executed concurrently on different machines.

Therefore, based on Table 2, we calculate the actual time by dividing the serial time by the concurrency factor. This eliminates time differences and better simulates the simultaneous execution of multiple shards. Table 2 lays the foundation for subsequent experiments on the impact of shard size on FL.

For parameter settings, we define the time efficiency factor (14):

(14) $$\frac{1}{n}\sum_{i=1}^{n}\frac{acc_{i}}{t_{i}}.$$

to calculate and obtain the most suitable learning rate for DSFL. With Equation (14), we can find a higher $acc_{i}$ learning rate for a shorter time interval $t_{i}$. We obtain a learning rate of 0.1 (with a time efficiency factor of 0.109), 0.01(0.234), 0.001(0.366), 0.0001(0.567), and 0.00001(0.530). The overall time efficiency factor increases initially and then decreases as the learning rate decreases. The optimal learning rate is 0.0001, where the time efficiency factor is highest. This indicates that DSFL can train better models in a shorter time with this learning rate.

As the learning rate decreases from 0.1 to 0.00001, the model’s stability improves, showing smaller fluctuations in accuracy. Regardless of the learning rate, the time efficiency factor increases in the later stages of training, indicating that the model improves faster as training progresses. Compared to 0.1, the learning rate of 0.0001 allows the model to converge quickly in the early stages while also achieving higher accuracy. Moreover, the number of global epochs in our research is 80, and the batch size is 32 because the model will definitely converge after 80 epochs. Parameters $\lambda_{1}$ and $\lambda_{2}$ are set to 0.3 to achieve a more balanced effect among the three random walk algorithms. During the training process, each point on DAG represents the accuracy of a transaction and reflects the global accuracy as well. Since the shards utilize a common test set, the evaluations also represent global accuracy.

#### 4.1.2. Datasets

We ensure that the total amount of data is the same, with each user having an equal amount of IID data. The experiments primarily use the following three datasets:

- CIFAR-10, consisting of 32 × 32 pixel images, is a popular benchmark for machine learning, containing 50,000 training and 10,000 test images.
- MNIST, featuring single-channel images of 28 × 28 pixels each, contains 60,000 training and 10,000 test images, all representing handwritten digits from 0 to 9.
- CIFAR-100, consisting of 32 × 32 pixel color images, is a widely used benchmark for machine learning, containing 50,000 training and 10,000 test images. It includes 100 classes, with 600 images per class, covering a broad range of objects from animals to vehicles.

The preprocessing for CIFAR involves randomly cropping images to 32 × 32 pixels while adding 4-pixel padding around the images, which helps improve the robustness of the model. Each channel is then normalized to have a mean of 0 and a variance of 1, achieved by calculating the mean and standard deviation from the training set.

Handling MNIST involves converting the single-channel images into three-channel images and normalizing the pixel values. This normalization shifts and scales the values, centering the data around 0 with a standard deviation of 1 by subtracting 0.5 from each pixel and dividing by 0.5.

#### 4.1.3. Baseline and Experimental Group Setup

In our experiments, we establish three baseline comparisons:

- Blockchain-FL [10]: In this scenario, all clients are in the same shard, effectively conducting synchronous training. This results in a single-chain structure in DAG, simulating a blockchain structure. It is important to note that this is not an actual blockchain because we continue to utilize DAG consensus algorithms. Traditional blockchain consensus typically requires at least 10 min to generate a block, which is inefficient. Therefore, we use this single-chain structure merely as a reference point.
- DAG-FL [14]: There is one client per shard, meaning that all clients interact directly with the DAG blockchain in an asynchronous manner, similar to DAG-FL. By comparing this direct asynchronous training without client shard integration, we can observe the advantages of the two-layer structure of DSFL, which combines synchronous and asynchronous training.
- Chains-FL [2]: The Chains-FL consists of a two-layer architecture, with the key feature of introducing DAG’s timeout pruning technique to ensure the freshness of the models. The DAG consensus relies solely on the accuracy of the validation tips and their ordering.

In experimental groups of DSFL, one group consists of 5 clients per shard, while the other group consists of 10 clients per shard. In this context, C = 2 represents two clients per shard.

### 4.2. Impact of Shard Size on the Performance of DSFL

**Figure 8:**
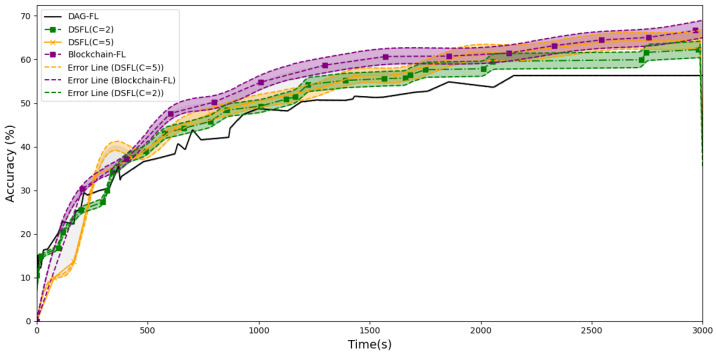
DSFL training accuracy with no latency for all clients.

**Figure 9:**
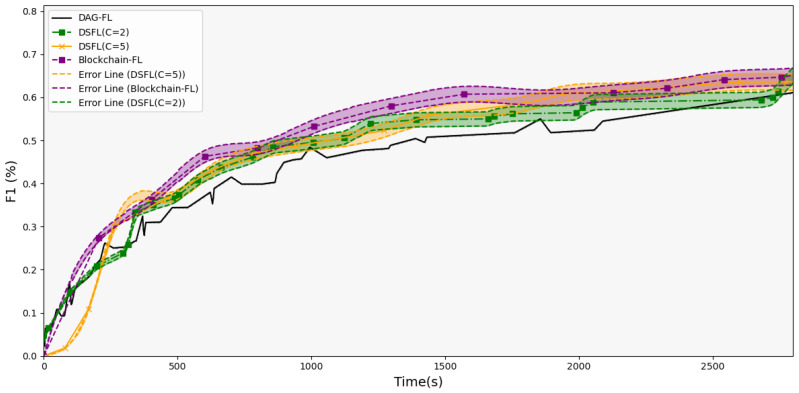
DSFL training F1 score with no latency for all clients.

We aim to observe the impact of adjusting the shard size of DSFL on the training process and to find a better shard size to improve FL efficiency. Meanwhile, we conduct a comparative analysis of the concurrency performance of DSFL and other baseline methods under ideal conditions. In essence, adjusting the shard size means altering the client grouping method while keeping the total data volume unchanged. The lines represent the global accuracy recorded on the DAG blockchain. In an ideal scenario with no delays for each client, the accuracy and F1 score are shown in Figure 8 and Figure 9. The error lines in the figure represent a 10% variation in the repeated experimental data, indicating the uncertainties of the model.

During the initial training phase, the large-scale concurrent training among clients is effective, as in DAG-FL and DSFL(C = 2), because clients interact more directly with DAG, and transaction nodes can be generated on DAG early on. Shards with more clients will need to wait for users to aggregate gradually. Although the overall training speed of Blockchain-FL may be slower and DAG grows at a reduced pace, the quality of training remains high. Due to the large number of users on each shard, the aggregated model achieves a more comprehensive effect. Therefore, Blockchain-FL and DSFL(C = 5) can converge more quickly in the later stages.

Meanwhile, DSFL groups outperform the pure asynchronous interactions of DAG-FL. Since each client’s data volume is small and the accuracy of the model is low, the submitted models are hard to integrate quickly into a better model within DAG. Sometimes, two suboptimal models might combine into an even worse model. However, there is no significant advantage compared to the Blockchain-FL. The overall trend shows that increasing the number of clients within a shard leads to better results. Ideally, if the training states of all clients are synchronized, efficiency will increase from purely asynchronous aggregation to fully synchronous. In a synchronized training state, each client’s model updates at the same pace. Synchronization allows all updates to combine into a unified, globally consistent model at each aggregation round. Synchronization reduces the bias and noise from inconsistent gradient updates, making the model more stable. In contrast, fully asynchronous aggregation keeps each client’s training state independent. This lack of synchronization adds bias, which can cause fluctuations or even conflicts. Hence, the synchronization of training states gradually enhances the convergence rate and overall performance of the model.

However, the experiments are conducted under the ideal condition of perfect synchronization among clients within all shards without any delays. In reality, achieving perfect synchronization among all clients is impractical, especially due to communication delays and time zone differences. Therefore, we conduct additional experiments simulating real-world conditions where each client experiences a time delay of 0–150 s in the local training phase.

**Figure 10:**
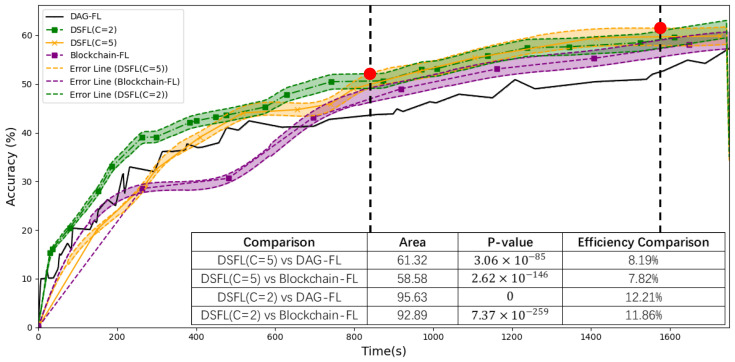
DSFL training accuracy with latency for all clients. (The red dots indicate a change in the optimal DAG size of the DSFL).

**Figure 11:**
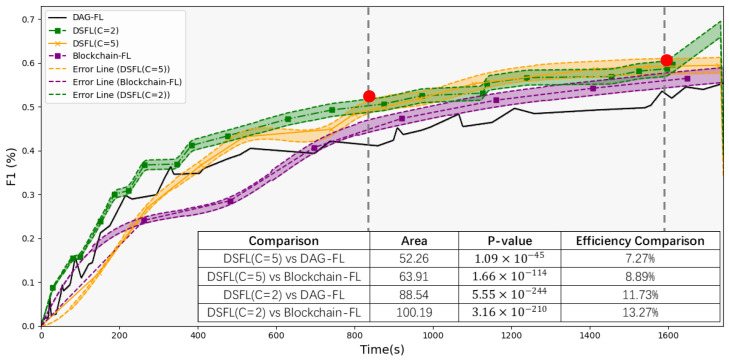
DSFL training F1 score with latency for all clients. (The red dots indicate a change in the optimal DAG size of the DSFL).

As shown in Figure 10 and Figure 11, under conditions where clients experience latency, the accuracy of DSFL shows an efficiency improvement of 8.19–12.21% compared to DAG-FL. The F1 score improves by 7.27% to 11.73%. Compared to Blockchain-FL, DSFL achieves an efficiency improvement of 7.82–11.86% in accuracy and 8.89–13.27% in F1 score. The area between the two curves represents the integral difference, with the size of the area reflecting the disparity between the two models. A larger area indicates a more significant difference. Statistical testing of the area difference yields very small *p*-values, such as 3.06 × 10^−85^, 2.62 × 10^−146^, 0, and so on. These *p*-values indicate that the differences between the models are highly significant and rule out the possibility of random chance.

DSFL combines the high integration of shard synchronization with the high concurrency of short-term multi-sharding. As the number of clients per shard C increases (which increases the maximum delay within each shard), the aggregation time for each shard becomes larger. Since the maximum delay across all shards determines the overall aggregation time, a larger shard size generally leads to a more significant overall aggregation time. As delays increase, the advantage of DSFL becomes more significant. Moreover, the accuracy and F1 score for the experimental groups exhibit a shift. Early DSFL(C = 2) has high concurrency, while later DSFL(C = 5) performs better. The high concurrency in the early stages comes from having more shards, which allows DSFL to handle a large number of tasks simultaneously. In the later stages, the system improves performance by increasing the load on each shard, reducing the number of shards, and lowering synchronization and communication costs.

Experiment 4.2 verifies the concurrency advantages of DSFL over DAG-FL and Blockchain-FL in environments with user latency and analyzes the optimal shard size at different time periods.

### 4.3. Effect of Shard Size on DAG Growth Patterns

**Figure 12:**
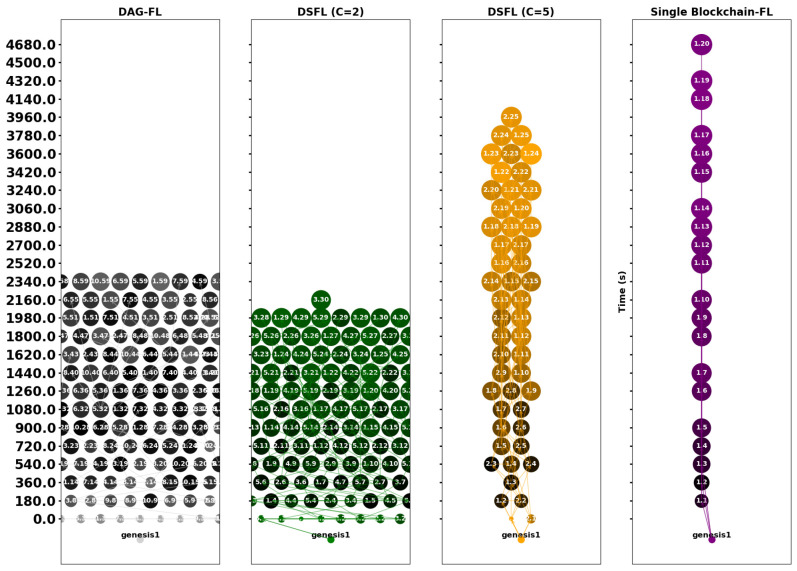
Simulation of the effect of shard size on DAG concurrency.

To explore the impact of shard size on the shape and growth speed of DAG, we compare the concurrency of DAG among the four groups. Figure 12 shows the concurrency of shards at various sizes, with the vertical axis scaled evenly to represent equal time intervals. Lighter colors indicate a higher F1 score.

Larger shards lead to a slower rate of DAG growth, as the publisher needs to wait longer to obtain better results from DAG. With fewer shards, the overall concurrency is not strong and approaches a single-chain structure more closely because fewer shards result in fewer parallel processing units, thus creating a bottleneck that limits throughput. Conversely, smaller shards allow for higher concurrency but yield lower training accuracy.

**Figure 13:**
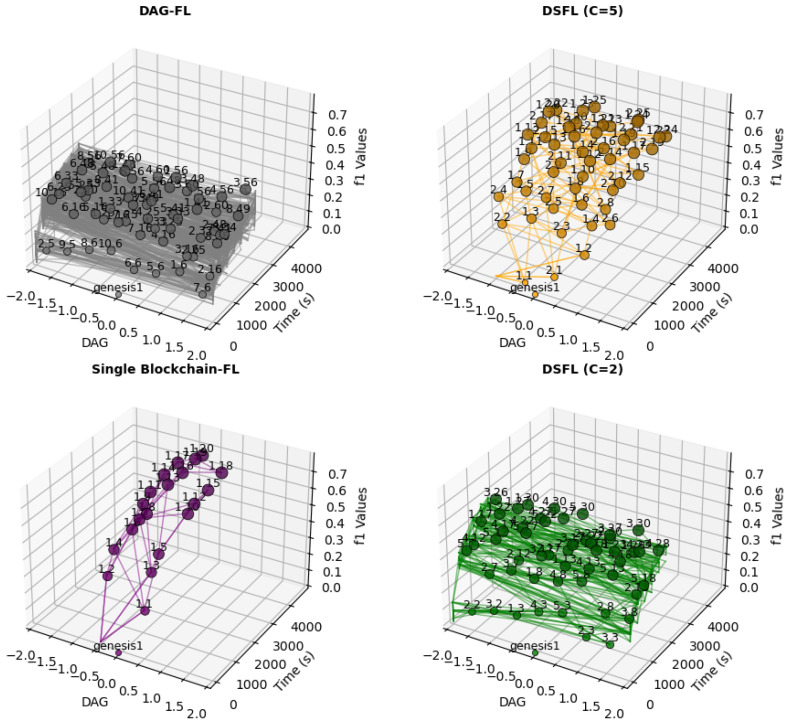
Three-dimensional view of DAG morphology and F1 score.

To provide a more intuitive representation of the F1 score, we incorporate the F1 score along the *Z*-axis in a three-dimensional graph, as shown in Figure 13. Although DAG-FL and DSFL(C = 2) exhibit large-scale concurrency, the visualization appears flatter. In contrast, the points become more concentrated in Blockchain-FL and DSFL(C = 5), yet the F1 score is higher. DAG-FL and DSFL(C = 2) can handle more parallel tasks. Since each client processes less data, the training samples are more scattered, leading to poorer performance. In Blockchain-FL and DSFL(C = 5), the computational load increases, and each client processes more data, improving the training performance and yielding a higher F1 score. Although concurrency is lower, and the overall parallel processing capacity of shards decreases, the nodes on DAG become more concentrated.

In conclusion, DAG-FL is suitable for scenarios requiring large-scale concurrency and high parallel processing capabilities. It is ideal when the focus is on completing many tasks simultaneously, but the trade-off of a lower F1 score is acceptable. DSFL is suitable for cases where high accuracy is prioritized, along with enhanced data processing per client. It works well in scenarios demanding a balance between accuracy and concurrency. Still, it leans towards achieving better training outcomes, such as the medical field requiring robust model performance and data privacy protection. Blockchain-FL fits scenarios where accuracy is critical, such as financial systems. The lower concurrency is acceptable in exchange for better training performance and higher F1 scores.

### 4.4. Comparative Experiments with Different Datasets

**Figure 14:**
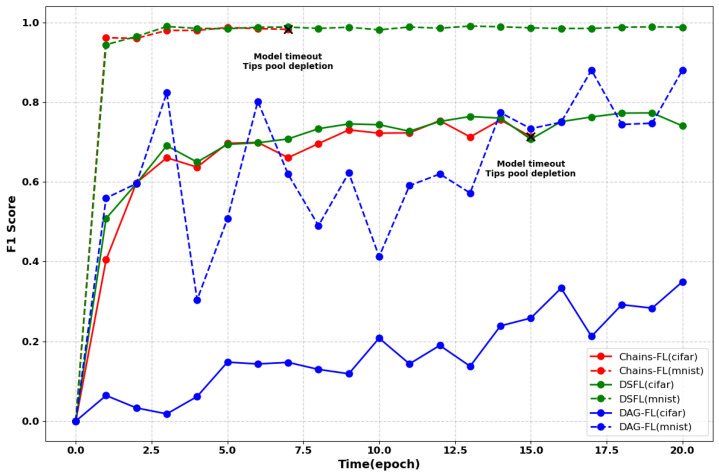
F1 score of the different methods on different datasets.

To compare the performance of DSFL, Chains-FL, and DAG-FL, we conduct experiments using the CIFAR and MNIST datasets. DSFL converges within the first 20 epochs. As shown in Figure 14, in fields with a small number of clients, high latency, and high data demands—such as the medical domain—the timeout pruning mechanism of Chains-FL may lead to a Tips pool depletion issue. Due to the limited data, models time out during the waiting period, resulting in a lack of valid transaction updates in the DAG, which interrupts the training tasks.

In contrast, DAG-FL suffers from low training accuracy and slower model convergence because of direct user interactions. DSFL addresses these challenges by eliminating outdated models through natural degradation via branching rather than pruning. This approach avoids resource depletion issues. Consequently, DSFL demonstrates robust performance in scenarios characterized by high latency, small sample sizes, and significant data demands.

### 4.5. Accuracy of Adaptive Algorithm

To better integrate the advantages of the three DAG consensus algorithms, we design an adaptive algorithm, which involves selecting different algorithms at different stages to enhance algorithm efficiency and control DAG concurrency.

**Figure 15:**
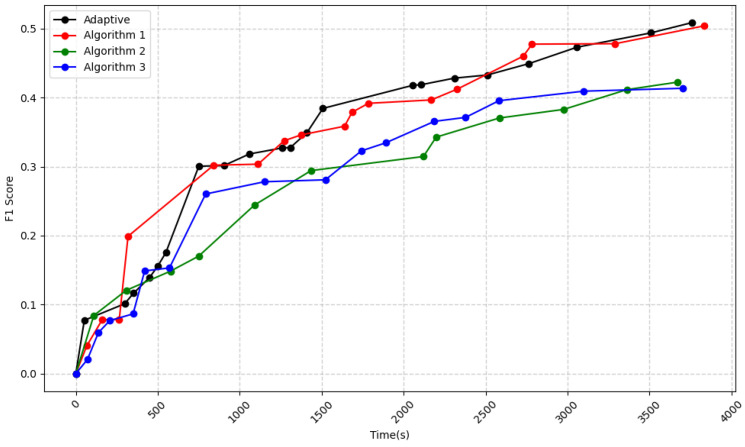
F1 score of the adaptive algorithm and three algorithms.

We select Algorithm 1 for the first 1500 s, and thereafter, we primarily use Algorithm 2. However, as shown in Figure 15, this shift in algorithm preference does not lead to a decline in the performance of the adaptive algorithm. Rather, it consistently outperforms the individual algorithms. Because the adaptive algorithm can adjust and select the most suitable algorithm for different periods, preventing the accumulation of disadvantages. Since the structure and branching of DAG involve continuously refining the model to form new paths, the adaptive algorithm is able to retain the advantages of the algorithm in the previous stage. Meanwhile, the adaptive algorithm incorporates both the high concurrency and accuracy characteristics of Algorithm 1 and the fairness aspects of Algorithm 2 in later stages, which will be discussed below.

### 4.6. Impact of the Adaptive Algorithm on the Fairness of the System

In addition to the high concurrency of Algorithm 1, we aim to investigate whether the adaptive algorithm can enhance the fairness of the DAG community. This requires studying the weight distribution of nodes in the DAG network to prevent degradation into a single chain and to avoid resource concentration on a few nodes forming a single path. Such a situation would lead to the issue where only a small number of shards with strong computing power and ample data would have the right to achieve global aggregation and receive tokens.

**Figure 16:**
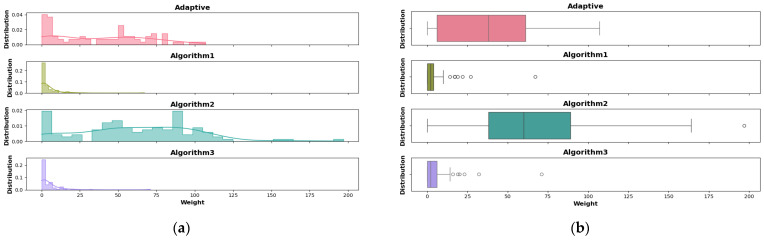
Comparison of fairness between the adaptive algorithm and three algorithms: (**a**) weight distribution graph; (**b**) box plot.

We compare the weight distribution of the adaptive algorithm with the three individual algorithms. As shown in Figure 16, Algorithms 1 and 3 exhibit a skewed distribution, indicating that the walking weights are overly concentrated, resulting in most nodes being unable to gain tokens. In contrast, Algorithm 2 facilitates a more uniform weight distribution within DAG. The slow verification process of Algorithms 1 and 3 leads to the obsolescence of models in certain branches, which causes a decrease in performance and the degradation of those branches. Eventually, this leads to the concentration of weights on a specific branch. In contrast, weight-based Algorithm 2 considers other branches where the parent nodes are located, thus distributing the weights more evenly. By combining the advantages of the three algorithms, the adaptive algorithm achieves a distribution closer to a normal distribution, ensuring that all nodes in the DAG network can receive incentives, thereby enhancing the fairness of the DAG community.

### 4.7. Comparative Experiments in Class Unbalanced Scenarios

In domains with high data demands and strict privacy requirements, such as healthcare, data are often class-imbalanced. Class imbalance can exacerbate data heterogeneity, posing additional challenges. To explore model performance under class-imbalanced scenarios and enhance its generalization capability, we construct an experimental setting with class imbalance, where the smallest class contains 2000 samples, and the largest class contains 5000 samples.

In Table 3, DSFL consistently outperforms the other methods in both scenarios, achieving an average accuracy of 15.19% in the imbalanced class scenario and 70.15% in the balanced class scenario. This indicates that DSFL is more robust to class imbalance and maintains strong generalization capabilities. The superior performance of DSFL can be attributed to its ability to handle heterogeneous and imbalanced data distributions effectively. Its design incorporates mechanisms to reduce the negative impact of data heterogeneity, such as more efficient handling of outdated models through natural degradation rather than strict pruning. This ensures that the training process remains stable, even when the data are highly imbalanced. Additionally, DSFL’s distributed consensus mechanism allows for more balanced participation of clients, which helps mitigate the effects of dominant classes in imbalanced datasets, leading to better generalization.

### 4.8. Datasets, Modeling, and Consensus Tuning Experiments

From Table 4, it is evident that the commission-shard consensus mechanism generally performs better than the raft-shard consensus mechanism. Due to its streamlined communication and election processes within shards, the commission-shard consensus mechanism is more efficient and reliable in handling large-scale networks and high-throughput demands. Additionally, both ResNet-18 and ResNet-34 demonstrate good accuracy within the DSFL framework. This indicates that our model is adaptable to various datasets and possesses strong generalizability.

### 4.9. Strengths, Limitations, and Future Improvements

Through experiments, we validate the advantages of our DSFL model in terms of high concurrency, fairness, and robustness, as shown in Table 5, but the experiments also reveal some aspects that we have not fully accounted for. For instance, our adaptive algorithm can not implement real-time automated selection. Additionally, our three lines of defense have not been designed with specific experiments to substantiate their security. Our primary focus is on enhancing efficiency and controlling concurrency. In the future, we will explore the applicability of DSFL under federated transfer learning and validate the model’s security using more privacy-sensitive datasets.

## 5. Conclusions

By establishing a Shard-DAG-Publisher tripartite task flow and training system, we achieve a cyclic task architecture to enhance the concurrency of traditional small-scale Blockchain-FL with slower consensus algorithms. At the same time, we ensure fair incentives for all clients through our random walk algorithm to maintain client participation rates.

Through eight experiments, we demonstrate DSFL’s advantages in high concurrency and fairness while adjusting the DAG size through concurrency control. In concurrency, DSFL improves accuracy by 8.19–12.21% and F1 score by 7.27–11.73% compared to DAG-FL. Compared to Blockchain-FL, DSFL shows an accuracy gain of 7.82–11.86% and an F1 score improvement of 8.89–13.27%. DSFL outperforms DAG-FL and Chains-FL on both balanced and imbalanced datasets. It achieves a balance between efficiency and fairness through a fusion of three DAG consensus algorithms and effectively mitigates malicious node attacks with a three-layer defense design. Comparisons of two shard consensus mechanisms show that commission-shard consensus performs better, especially in scenarios with larger shard sizes and more clients.

In the future, we plan to further explore derivative issues introduced by DAG structures, along with a comprehensive examination of Byzantine fault tolerance and the impact of malicious nodes within DSFL frameworks. Our continued research aims to address these challenges to enhance the security and flexibility of distributed systems.

## Figures and Tables

**Table 1:** Comparison of DSFL and related research.

Related Research	Main Problem	Specific Content	DSFL Improvement
Blockchain-FL [10]	Low concurrency	A block mined every 10 min	Synchronization within shards and asynchronous implementation on the DAG blockchain
Chains-FL [2]	Not suitable for high latency training tasks	Risk of tips pool depletion	
DAG-FL [14]	Low efficiency	A large number of clients interacting asynchronously with DAG leads to low global accuracy	
PPFL [7]	Computationally complex	HE (Homomorphic Encryption) is computationally complex and costly	
An Incentive-aware Blockchain-enabled FL Platform [11]	Unfair incentive	Clients with small sample sizes and insufficient information may have a low probability of spontaneously selecting high-performing models	DSFL combines three random walk algorithms to develop an adaptive approach, resulting in a fairer incentive mechanism and encouraging greater client participation.
FGFL [12]	Unfair incentive	Nodes with lower computational power and higher latency may fail to receive incentives	

**Table 2:** Time conversion: when using serial processing to simulate parallel execution, the time increases as the number of shards grows.

10 Clients/Shard	Average time	1 Shard with 10 Clients each	Average time	1.0000 times
	234.8291		234.8291	
5 Clients/Shard	Average time	2 Shards with 5 Clients each	Average time	1.6328 times
	161.5541		259.0168	
2 Clients/Shard	Average time	5 Shards with 2 Clients each	Average time	4.8331 times
	72.3809		349.8216	
1 Client/Shard	Average time	10 Shards with 1 Client each	Average time	10.1084 times
	41.1607		416.0720	

**Table 3:** Performance comparison in scenarios of balanced vs. imbalanced data classes.

Average Accuracy	Imbalanced Class Scenario	Balanced Class Scenario
DSFL	15.19%	70.15%
Chains-FL	14.48%	67.31%
DAG-FL	7.41%	19.91%

**Table 4:** Experiments with different models and datasets in DSFL.

Datasets	Models	Consensus	Accuracy (%)	F1 Score
mnist	Resnet-18	raft	98.94	0.989
	Resnet-18	commission	99.09	0.991
	Resnet-34	raft	98.80	0.988
	Resnet-34	commission	99.03	0.990
cifar10	Resnet-18	raft	81.02	0.809
	Resnet-18	commission	78.69	0.786
	Resnet-34	raft	77.51	0.772
	Resnet-34	commission	78.49	0.783
cifar100	Resnet-18	raft	78.23	0.781
	Resnet-18	commission	79.79	0.796
	Resnet-34	raft	76.67	0.765
	Resnet-34	commission	81.12	0.811

**Table 5:** Comparison of methods (√ indicates a feature is implemented, × indicates it is not).

	High Concurrency	Efficiency	Fair Incentive Mechanism	Security
Blockchain-FL [10]	×	√	√	×
DAG-FL [14]	√	×	×	×
Chains-FL [2]	×	√	×	Risk of tip pool depletion
DSFL	√	√	√	√

## Data Availability

The data in this study are public. Cifar-10: http://www.cs.toronto.edu/~kriz/cifar.html (accessed on 9 May 2024) MNIST: https://yann.lecun.com/exdb/mnist/ (accessed on 9 May 2024) Cifar-100: https://www.cs.toronto.edu/~kriz/cifar.html. (accessed on 9 May 2024).

## Notations

Summary of main notations.

**Notations**	**Meaning**	**Notations**	**Meaning**
t	Tip	D	Dataset
Txn	Transaction	$M_{j}$	Model j
C	Client	acc	Accuracy of the models
N	Node	$A^{\left(t\right)}$	A total accumulator at a certain time
w	Weight	$\lambda_{1}$,$\lambda_{2}$	Coefficient used to adjust tip selection algorithms
$s_{i}$	Shard information of $C_{i}$	p	The number of wandering rounds
${Term}_{r}$	The requested term	T	The trace set of a tip walking by
${Term}_{C}$	The current term	A	The set of approvers of a node
$L_{i}$	The log for node i	T	The tangle
$I_{prev}$	The index of the previous log entry	G	The Genesis node
$I_{last}$	The last index applied to the state machine by node i	$S_{max}$	The maximum number of steps to prevent wandering into a deadlock
$I_{i}$	The index that node i has committed	$V_{i}$	Set of transactions accessed by the i-th trajectory
Com	Committee		
